# Examining Recruitment Strategies in the Enrollment Cascade of Youth Living With HIV: Descriptive Findings From a Nationwide Web-Based Adherence Protocol

**DOI:** 10.2196/40077

**Published:** 2023-04-12

**Authors:** Sitaji Gurung, Stephen Scott Jones, Kripa Mehta, Henna Budhwani, Karen MacDonell, Marvin Belzer, Sylvie Naar

**Affiliations:** 1 Department of Health Sciences New York City College of Technology The City University of New York Brooklyn, NY United States; 2 Department of Psychology Hunter College The City University of New York New York, NY United States; 3 College of Nursing Florida State University Tallahassee, FL United States; 4 Department of Behavioral Sciences and Social Medicine, Center for Translational Behavorial Science College of Medicine Florida State University Tallahassee, FL United States; 5 Children’s Hospital Los Angeles Los Angeles, CA United States

**Keywords:** recruitment methods, enrollment challenges, digital technology, adherence protocol, youth living with HIV, COVID-19

## Abstract

**Background:**

Digital strategies and broadened eligibility criteria may optimize the enrollment of youth living with HIV in mobile health adaptive interventions. Prior research suggests that digital recruitment strategies are more efficient than traditional methods for overcoming enrollment challenges of youth living with HIV in the United States.

**Objective:**

This study highlights the challenges and strategies that explain screening and enrollment milestones in a national web-based adherence protocol for youth living with HIV.

**Methods:**

Baseline data from a national web-based HIV adherence protocol for youth living with HIV, collected from July 2018 to February 2021, were analyzed. A centralized recruitment procedure was developed, which used web-based recruitment via *Online Master Screener*; paid targeted advertisements on social media platforms (eg, Facebook and Reddit) and geosocial networking dating apps (eg, Grindr and Jack’d); and site and provider referrals from Subject Recruitment Venues and other AIDS service organizations, website referrals, and text-in recruitment.

**Results:**

A total of 3 distinct cohorts of youth living with HIV were identified, marked by changes in recruitment strategies. Overall, 3270 individuals consented to screening, 2721 completed screening, 581 were eligible, and 83 completed enrollment. We examined sociodemographic and behavioral differences in completing milestones from eligibility to full enrollment (ie, submitting antiretroviral therapy and viral load data and completing the baseline web-based survey). Those with the most recent viral load tests >6 months ago were half as likely to enroll (odds ratio 0.45, 95% CI 0.21-0.94). Moreover, eligible participants with self-reported antiretroviral therapy adherence (SRA) between 50% and 80% were statistically significant (*P*<.001 to *P*=.03) and more likely to enroll than those with SRA >80%.

**Conclusions:**

The findings add to our knowledge on the use of digital technologies for youth living with HIV before and during the COVID-19 pandemic and provide insight into the impact of expanding eligibility criteria on enrollment. As the COVID-19 pandemic continues and the use of and engagement with social media and dating apps among youth living with HIV changes, these platforms should continue to be investigated as potential recruitment tools. Using a wide variety of recruitment strategies such as using social media and dating apps as well as provider referral mechanisms, increasing compensation amounts, and including SRA in enrollment criteria should continue to be studied with respect to their ability to successfully recruit and enroll eligible participants.

**International Registered Report Identifier (IRRID):**

RR2-10.2196/11183

## Introduction

### Background

HIV continues to have a disproportionate impact on adherence, and youth living with HIV aged 15-24 years have been shown to be poorly adherent to antiretroviral therapy (ART) [1]. Linkage to care is a critical step for youth who test positive for HIV in successful HIV care and treatment and is typically defined as the completion of a first medical clinic visit after HIV diagnosis [2]. Once youth living with HIV are linked to care, the next steps are crucial for maintaining adherence to ART and sustaining viral suppression. More than half of adults achieve viral suppression; however, only approximately 16% of youths living with HIV are virally suppressed at 5 years of diagnosis [3]. To curb the growing HIV epidemic among youth, it is essential to maintain contact with eligible study participants and engage in regular HIV care or adhere to HIV treatment regimens [4]. However, recruiting youth living with HIV in the treatment cascade is challenging [2,5] because of several factors, including transportation barriers in rural areas [6,7] and the lack of access to health services and technology [8]. Although traditional recruitment methods such as field-based strategies and venue-based sampling have been adapted for adults living with HIV [9,10], such strategies may not be efficient or acceptable methods of recruitment for rural and clinic-based populations and web-based samples. A nationwide web-based adherence protocol is needed to recruit youth that represent rural populations and those who may be hard to reach through traditional sampling strategies [8].

Researchers have started using social media platforms to recruit high-risk HIV populations and hard-to-reach populations [11] and for web-based recruitment of youth for mobile health interventions of the National Institutes of Health–funded Adolescent Medicine Trials Network for HIV/AIDS Interventions (ATN) [12]. The use of web-based recruitment strategies such as paid recruitment advertisements on social media platforms and geosocial networking dating apps have been established. A systematic review that included 110 unique studies found that Facebook is an effective and cost-efficient recruitment strategy to target stigmatized populations including youth living with HIV [13]. Dating apps such as Grindr were found to be successful advertising campaigns for recruiting young men who have sex with men (MSM) for mobile health studies when compared with other recruitment efforts [12]. A randomized controlled trial that used a clinic-based recruitment strategy to examine the effectiveness of a technology-enhanced community health nursing intervention was found to improve medication adherence and viral suppression among youth living with HIV [2]. However, the additional effort required by clinic staff makes web-based recruitment a more effective strategy for recruiting youth living with HIV based on sexual orientation and gender [8]. Although these studies have focused on the recruitment of youth populations with minority identities (ie, racial and ethnic minority groups or sexual and gender minority groups), young women at higher risk for HIV (ie, those who are unaware of their partner’s risk factors or those who have been exposed to intimate partner violence) are underrepresented in adaptive ART adherence interventions [14,15].

Recruitment challenges have been reported as a major factor contributing to the premature termination of clinical trials, as described in previous studies [16,17]. Recruiting youth living with HIV is particularly challenging for adaptive interventions in which the dosage of the intervention is adjusted based on the participant’s response [6,18], such as interventions adapted using a Sequential Multiple Assignment Randomized Trial (SMART) design [18] in HIV treatment protocols [19]. SMART trial participation requires multiple intervention stages, blood panels, and substantial effort from the participant. Participant recruitment and completion of multiple steps, including submissions of proof of ART prescriptions and viral load (VL), providing release of information from the primary HIV clinic, and completing the web-based survey, are critical components of enrollment in treatment protocols. However, to date, there is limited research examining approaches and strategies for the successful recruitment of US adolescents, including young women, into treatment protocols. The visual analog scale (VAS), used as a self-reported ART adherence (SRA) measurement, has been recommended in clinical practice and research design [20,21]. In light of the current COVID-19 pandemic, which has introduced pressure from health care infrastructure to provide sufficient remote services, leveraging advances in technology (eg, VAS) and digital media may offer novel recruitment methods.

Successful recruitment and enrollment of eligible study participants are crucial for conducting human subjects research [22]. Researchers use various approaches to reach the target population, and recruitment may be less challenging when the target population is broad (eg, adults of any race). However, when inclusion criteria are narrow or when working with stigmatized groups, recruitment can be more difficult [23]. Conducting HIV outcomes and behavioral research with youth living with HIV and those with other stigmatized identities is of high public health priority [24]. These studies have significant potential to make a positive impact on public health. Improved adherence to ART can lead to viral suppression, which in turn can result in better health outcomes for young people and a decrease in HIV transmission [25,26]. However, to conduct statistically powered, scientifically rigorous HIV studies with youth living with HIV, researchers must be able to recruit and enroll this often stigmatized population [27]. Considering these factors, we conducted a methods-focused exploratory study to elucidate youth living with HIV–friendly and acceptable recruitment and enrollment strategies within a national web-based HIV adherence protocol.

### Objectives

The primary purpose of this study was to explore strategies that explain screening and enrollment milestones in a national web-based adherence protocol for youth living with HIV. The following questions guide this study:

1. What recruitment strategies influence screening and enrollment milestones? Do web-based recruitment strategies (eg, social media platforms and geosocial networking dating apps) and provider-based referral mechanisms (eg, ATN providers and Ryan White providers) play a vital role in completing milestones from eligibility to full enrollment?
2. What compensation strategies influence screening and enrollment milestones? Does higher compensation or incentivizing full completion by building bonus structures play a vital role in completing milestones from eligibility to full enrollment?
3. Describe the study eligibility criteria for recruiting youth living with HIV. Does the expansion of eligibility criteria to include self-reported adherence measure influence screening and enrollment milestones?

## Methods

### Overview

ATN 144 SMART is an adaptive antiretroviral adherence intervention designed to improve self-management and maintain VL suppression for youth nonadherent to ART while understanding the context for wide-scale implementation in an effectiveness-implementation type 1 hybrid trial [19]. To reach a broader audience beyond the ATN sites, SMART developed a centralized recruitment process that uses (1) web-based recruitment via *Online Master Screener* (OMS); (2) paid targeted advertisements on social media platforms (eg, Facebook and Reddit) and geosocial networking dating apps (eg, Grindr and Jack’d); and (3) site and provider referrals from *Scale It Up* Subject Recruitment Venues (SRVs) [28] and other AIDS service organizations outside of the ATN [29], website referrals, and text-in recruitment.

### Recruitment Strategies

Recruitment was conducted between July 2018 and February 2021. Potential participants were routed to the SMART study through the OMS, which determined the eligibility for several studies. If the interested individuals were potentially eligible for SMART, a screen was displayed to inform them that they might be eligible for the study, followed by a separate web-based form to collect contact information. The study shifted to paid national advertising campaigns on social media sites to boost their reach and engagement with targeted audiences. Filtering features were used while setting up the web-based ad campaigns to target social media users who indicated in their profiles that they were aged between 15 and 24 years and lived in the United States. Other *interest* categories were used to target individuals based on social media site algorithms, which would identify young MSM or young men and women who have interacted with HIV-related pages and status messages on social media platforms. We also recruited through advertisements on geosocial networking dating apps. Two methods of advertisements were used to recruit participants: (1) a pop-up message shown when a user first logs in, encouraging users to click through to determine eligibility for the study, and (2) a message sent by the app directly to the user’s inbox. Interested participants clicked the advertisement and were routed to the study screener to determine eligibility and learn more information on how to enroll in the study.

To diversify recruitment and reach individuals outside of a web-based setting, the protocol team identified AIDS service organizations or clinics including Ryan White–funded recipients and other community-based organizations where potential participants can learn about the study. We set up a keyword using Trumpia [30], a popular text messaging marketing service. Interested individuals texted a keyword (eg, *RESEARCH*) to a 5-digit number (eg, 99-000) to learn about how to screen for the study. Once a potential participant sent the keyword to the 5-digit number, they received an automated text message with a link to a landing page, where they could complete the preliminary screener specific to this study. We downloaded the listserve data from the *Health Resources and Services Administration* (HRSA) website [29]. By accessing the HRSA website, the email addresses of Ryan White recipients were filtered using Ryan White as the *Grant Program Name* on the *Active Grants* Data Downloads page. We also launched a website on which providers outside of the ATN network can learn about the SMART study and request recruitment materials or further information. Outreach emails were sent to 545 Ryan White–funded recipients, with links to the website and recruitment materials order form; we received 32 orders for recruitment materials. Flyers and other recruitment materials with a text-in number were distributed to SRVs and other clinics that provide care for youth living with HIV.

### Ethics Approval

The study was approved by the institutional review board (IRB) at Florida State University (approval STUDY00000548) and performed in accordance with the ethical standards laid down in the 1964 Declaration of Helsinki and its later amendments or comparable ethical standards. A signed and dated statement that the study materials were approved by the IRB was provided to each participating SRV before site initiation occurred. A reliance agreement was received from each SRV, confirming their agreement to conduct the study in accordance with the IRB approval. Electronic informed consent or assent (for those aged <18 years) and the Health Insurance Portability and Accountability Act of 1996 were obtained from each participant before any study-related procedures were performed. To further protect the privacy of the study participants, the study team obtained a certificate of confidentiality from the US Department of Health and Human Services.

### Participants and Enrollment Procedures

Participants were deemed eligible if they (1) were aged 15-24 years at the time of signing the informed consent or assent form, (2) were willing to provide proof of VL ≥200 copies/mL or blood specimens for HIV VL measurement, (3) were prescribed an ART medication regimen for a minimum of 3 months before screening for VL, (4) had a VL ≥200 copies/mL within 6 months before baseline enrollment, (5) were the sole owner of a device capable of sending and receiving calls and SMS text messages, and (6) were able to provide consent to the research team to communicate with the participant’s HIV care provider team.

Multiple challenges in enrollment resulted in 3 identifiable waves of cohorts. Each cohort was associated with various changes to the protocol and recruitment strategies to increase enrollment. Cohort 1 comprised initial enrollment between July 10, 2018, and June 19, 2019. To ensure that our target population found the protocol desirable, we made multiple revisions to the recruitment scripts and framed the study description in such a way that their participation would contribute to the scientific community. With these changes being implemented, enrollment continued to be a challenge. We identified that the submission of VL results appeared to be a barrier to enrollment. To address this issue, we adapted the study eligibility criteria as a recruitment strategy, resulting in cohort 2.

Cohort 2 enrollment occurred between June 20, 2019, and January 20, 2020. First, in June 2019, the protocol team added SRA of ≤80% in the past 30 days to the original criteria to widen our net and changed the VL requirement timeline from 6 months to 12 months before baseline enrollment. However, these adaptations were not sufficient to increase enrollment. We then developed a plan to revise the participant compensation structure as a recruitment strategy to better match participant effort, which resulted in cohort 3.

Cohort 3 enrollment occurred between January 21, 2020, and February 24, 2021. Participant compensation was increased from US $200 (ie, US $40 for completing each assessment) to as much as US $500 (ie, US $60 for completing each assessment with US $10 increased compensation over time, US $10 bonus for completing the web-based survey within 1 week, and US $10 bonus for submitting the VL data within 2 weeks) because of the recognition of participant burden related to the multiple steps involved in enrollment. These steps included providing proof of ART prescriptions, obtaining VL measurements either at their home clinic or at Quest laboratories, granting us release of information from their primary HIV clinic, and filling out the web-based survey. In addition, to improve retention, the team agreed to escalate compensation at each 3-month assessment to support a high retention percentage to meet the required sample for successful analyses.

### Measures

During screening, participants were asked to self-report their age, ethnicity and race, gender identity, sexual identity, zip code (which was converted to regions), how they learned about the study (which was converted to recruitment source), and HIV outcome values (ie, most recent VL test, most recent VL result, last detectable VL test, ART prescribed, and first prescribed ART). The VAS was used to assess the percentage of HIV medications taken in the past 30 days on a scale from 0% to 100% [21,31]. This single-item scale was used to assess the revised enrollment criteria, that is, SRA of ≤80%.

### Statistical Analyses

Using SPSS (version 28; IBM Corp) and Mplus (version 8; Muthén and Muthén; a statistical modeling program), analyses were conducted to explore (1) if web-based strategies (eg, social media and geosocial networking data apps) and provider-based referral mechanisms (eg, ATN providers, Ryan White providers, and Northwestern University) had different recruitment outcomes, (2) if the expansion of eligibility criteria influenced recruitment, and (3) if adjusted compensation strategies (ie, increased compensation) influenced enrollment. Data were partitioned into cohorts to enable the analyses of recruitment and enrollment outcomes.

In addition, the analyses addressed questions of bias in sample composition introduced at each enrollment milestone. Descriptive statistics provided a quantitative foundation to compare the data for each recruitment source, sociodemographic identity, and HIV-related characteristic. Chi-square analyses compared group frequencies at each step of enrollment: (1) the submission of contact information when found to be eligible for screening, (2) submission of VL and proof of ART prescription, and (3) completion of the baseline survey. A multivariable logistic regression model was used to estimate the relative influence of sociodemographic data and HIV-related characteristics to predict full enrollment among eligible participants who submitted contact information across all cohorts.

## Results

### Overview

**Figure 1:**
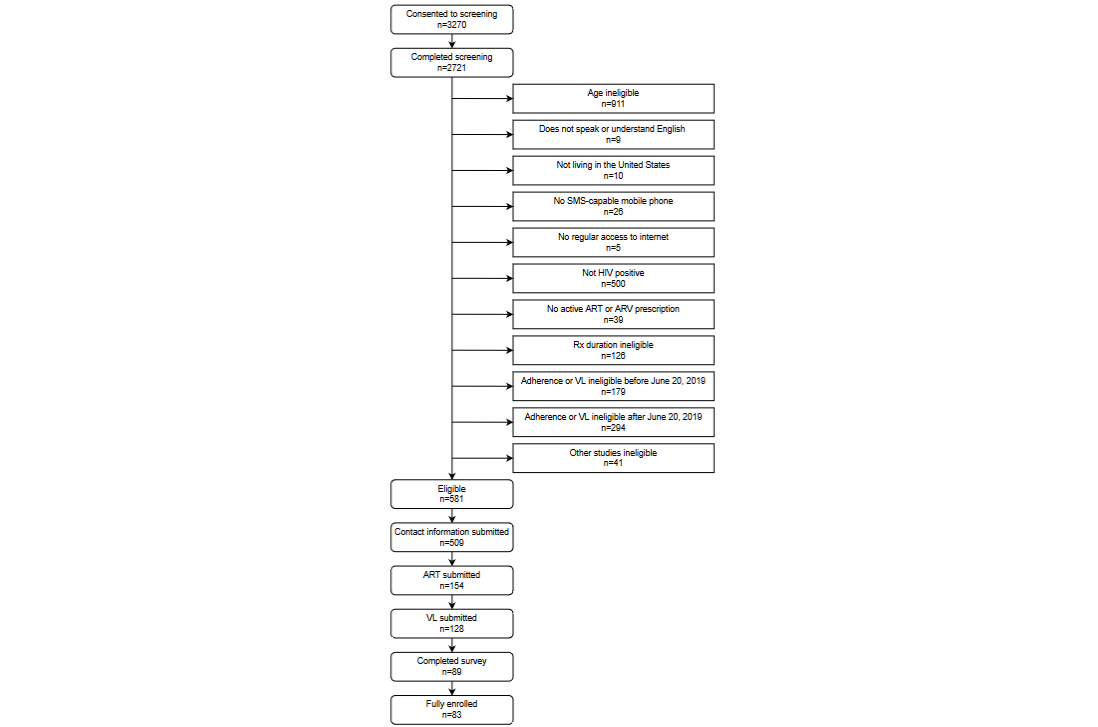
Sequential Multiple Assignment Randomized Trial screened flowchart. ART: antiretrovial therapy; ARV: antiretroviral; Rx: prescription; VL: viral load.

**Figure 2:**
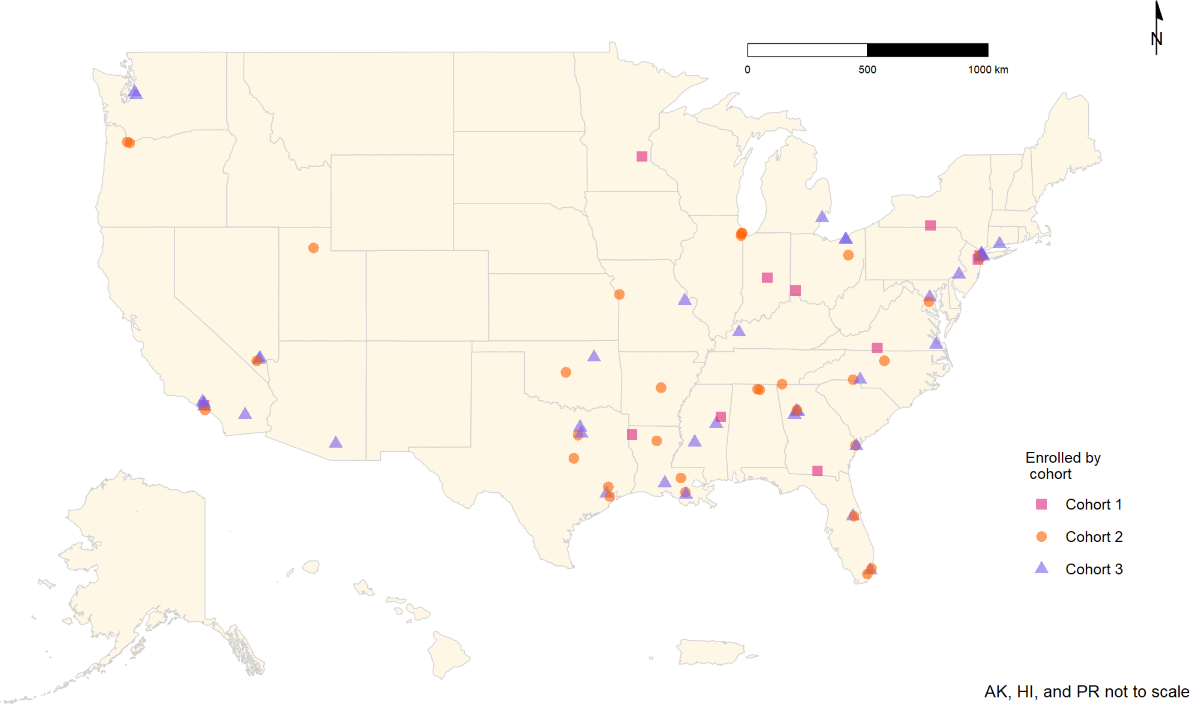
Geographic characteristics of the enrolled sample. AK: Alaska; HI: Hawaii; PR: Puerto Rico.

The flowchart in Figure 1 displays the progression of the participants from screening to enrollment. Figure 2 shows the geographical distribution of the enrolled participants. Table 1 displays the demographic characteristics and HIV outcomes for those screened for eligibility and the final enrolled sample.

**Table 1:** Demographics and clinical characteristics of screened and fully enrolled sample (N=2721).

Characteristic				Completed screening (N=2721), n (%)		Fully enrolled (n=83), n (%)
**Age (years)**						
	13-17			76 (2.79)		2 (2.41)
	18-20			398 (14.63)		13 (15.66)
	21-24			1338 (49.17)		68 (81.93)
	≥25			909 (33.41)		N/A^a^
**Race and ethnicity**						
	Black or African American			1168 (42.93)		46 (55.42)
	Hispanic			595 (21.87)		18 (21.69)
	White			593 (21.79)		10 (12.05)
	Other			365 (13.41)		9 (10.84)
**Region**						
	Northeast			471 (17.31)		11 (13.25)
	Midwest			416 (15.29)		12 (14.46)
	South			1244 (45.72)		44 (53.01)
	West			580 (21.32)		16 (19.23)
	Military, Puerto Rico			6 (0.22)		N/A
	Invalid zip			4 (0.15)		N/A
Metropolitan residence				1920 (70.56)		61 (73.49)
**Sexual identity**						
	Gay			1911 (70.23)		63 (75.9)
	Straight			174 (6.39)		9 (10.84)
	Bisexual			510 (18.74)		11 (13.25)
	Queer or other sexual identity			126 (4.63)		N/A
**Gender identity**						
	Man			2412 (88.64)		65 (78.31)
	Woman			107 (3.93)		11 (13.25)
	Nonbinary			202 (7.42)		7 (8.43)
**Recruitment source**						
	**Paid advertisement**			2320 (85.26)		48 (57.83)
		Social media (Facebook)	421 (15.47)		20 (24.09)	
		Networking apps (Grindr and Jack’d)	1844 (67.77)		28 (33.73)	
		Reddit	55 (2.02)		N/A	
	**Unpaid recruitment**			401 (14.74)		35 (42.17)
		Text in	75 (2.76)		4 (4.82)	
		Northwestern University	13 (0.48)		1 (1.2)	
		Online Master Screener or website	112 (4.17)		12 (14.46)	
		Other source	201 (7.39)		18 (21.69)	
**Most recent VL^b^ test**						
	<1 month ago			636 (23.37)		24 (28.92)
	2-3 months ago			738 (27.12)		33 (39.76)
	4-6 months ago			320 (11.76)		22 (26.51)
	7-12 months ago			110 (4.04)		4 (4.82)
	>1 year ago			76 (2.79)		N/A
	HIV negative or unknown			841 (30.91)		N/A
**Most recent VL result**						
	Undetectable (<200 copies/mL)			1020 (37.49)		36 (43.37)
	Detectable (at least 200 copies/mL)			317 (11.65)		21 (25.3)
	Unsure			543 (19.96)		26 (31.33)
	HIV negative or unknown			841 (30.91)		N/A
**Last detectable VL test**						
	<3 months ago			484 (17.79)		24 (28.92)
	4-6 months ago			229 (8.42)		17 (20.48)
	7-12 months ago			194 (7.13)		7 (8.43)
	>1 year ago			454 (16.69)		17 (20.48)
	Never detectable			128 (4.70)		4 (4.82)
	Unsure			391 (14.37)		14 (16.87)
	HIV negative or unknown			841 (30.91)		N/A
**ART^c^ Prescription**						
	Never prescribed			57 (2.09)		N/A
	Formerly prescribed			104 (3.82)		2 (2.41)
	Currently prescribed			1719 (63.18)		81 (97.59)
	HIV negative or unknown			841 (30.91)		N/A
**First prescribed ART**						
	<3 months ago			179 (6.58)		N/A
	3-6 months ago			184 (6.76)		11 (13.25)
	7-12 months ago			211 (7.75)		14 (16.87)
	>1 year ago			1249 (45.90)		58 (69.88)
	Never prescribed ART/HIV negative or unknown			898 (33.00)		N/A
**Self-reported adherence**						
	SRA^d^ ≤80%			775 (28.48)		75 (90.36)
	SRA >80%			1048 (38.52)		8 (9.64)
	Never prescribed ART/HIV negative or unknown			898 (33.00)		N/A
Cohort 1: initial enrollment				379 (13.93)		11 (13.25)
Cohort 2: SRA eligibility				329 (12.09)		32 (38.55)
Cohort 3: compensation increase				2013 (73.98)		40 (48.19)

^a^N/A: not applicable.

^b^VL: viral load.

^c^ART: antiretroviral therapy.

^d^SRA: self-reported antiretroviral therapy adherence.

### Screening and Enrollment Comparison of Cohorts

Screener responses collected from July 10, 2018, to June 19, 2019 (345 days) constituted cohort 1. During this period, 379 responses were collected for the original compensation scheme and eligibility criteria, which accounted for 13.93% (379/2721) of all the screeners completed in the study (Table 1). During this period, 12.9% (49/379) of eligible contacts were identified from those screened, with 32% (16/49) of eligible contacts submitting VL and proof of ART and 28.6% (14/49) of eligible contacts completing the baseline survey (Tables 2 and 3). The recruitment efforts in cohort 1 yielded 11 (22%) enrolled participants from 49 of the eligible contacts. Of the 11 participants enrolled, 8 (72%) were recruited from paid advertisements, with 3 (27%) from social media and 5 (45%) from networking apps. Unpaid recruitment yielded the 3 remaining enrollees from the 11 participants, with 1 (9%) enrollee from the OMS connected to our research laboratory webpage, 1 (9%) enrollee from a provider-supported text-in option for screening, and 1 (9%) enrollee referred by multiple-study recruitment surveys.

Cohort 2 spanned 214 days from June 20, 2019, to January 20, 2020, during which the eligibility criteria were adjusted and 329 completed screener responses were collected (Table 2). The eligibility rate more than doubled when compared with cohort 1, with 43.5% (143/329 of completed screeners) eligible respondents leaving contact information. Of the 143 eligible contacts, 44 (30.8%) had submitted VL and proof of ART, 33 (23.1%) completed the baseline survey, and 32 (22.4%) were enrolled. Of the 32 participants enrolled, 17 (53%) came from paid advertisements, with 9 (28%) enrollees from social media and 8 (25%) from networking apps. Of the 32 enrollees, an additional 15 (46%) enrollees came from unpaid recruitment efforts, with 8 (25%) from pooled recruitment surveys, 3 (9%) from a provider-supported text-in option, 3 (9%) from the OMS, and 1 (3%) from SMART Northwestern University study referral. As expected, the adjustment in eligibility criteria increased the proportion of eligible contacts from 12.9% (49/379) to 43.5% (143/329), whereas the number of enrolled tripled from 11 to 32. The proportion of enrolled participants remained constant, with 22.4% (32/143) of eligible contacts enrolled in both cohorts.

Participants screened from January 21, 2020, to February 24, 2021, comprised cohort 3; in these 400 days, compensation increased, and paid advertisements boosted recruitment efforts, with 2013 completed screeners yielding 15.7% (317/2013) of eligible contacts (Table 2). Of the 317 eligible contacts, 58 (18.3%) submitted VL and proof of ART, 51 (16.1%) completed the baseline survey, and 40 (12.6%) were enrolled. Of the 40 enrollees, paid recruitment yielded 23 (57%) enrollees, with 8 (20%) respondents from social media and 15 (37%) respondents from networking apps. Of the 40 enrollees, unpaid recruitment yielded 17 (42%) enrollees, with 8 (20%) enrollees from the OMS and 9 (22%) enrollees from pooled recruitment surveys.

Web-based (paid) recruitment efforts proved much more successful in cohort 1 than provider-based (unpaid) efforts, with 72% (8/11) of participants enrolled via web-based recruitment efforts versus 27% (3/11) from providers. This difference decreased in cohorts 2 and 3, with 53% (17/32) enrolled from web-based efforts and 46% (15/32) from provider referral efforts in cohort 2 and 57% (23/40) enrolled from web-based efforts and 42% (17/40) from provider referral efforts in cohort 3 (Tables 4 and 5).

Although the number of potential participants radically increased from 379 in cohort 1 and 329 in cohort 2 to 2013 in cohort 3, the proportion of eligible contacts was 15.74% (317/2013) of those screened in cohort 3, a decrease from 43.5% (143/329) in cohort 2 to a level similar to that observed in cohort 1 (49/379, 12.9%). Cohort 3, defined by enhanced compensation structures, proved less successful in encouraging eligible contacts to submit VL and proof of ART. The proportions of those who submitted VL and proof of ART were the lowest of the 3 cohorts, decreasing from 32% (16/49) in cohort 1 and 30.8% (44/143) in cohort 2 to 18.3% (58/317) in cohort 3. In addition, increased compensation appeared not to encourage the completion of the baseline survey. The completion of the baseline survey decreased from 28% (14/49) in cohort 1 and 23.1% (33/143) in cohort 2 to 16.1% (51/317) in cohort 3. Although the proportion of enrolled participants dropped to 12.6% (40/317) in cohort 3—approximately half that of the 22% (11/49) in cohort 1 and 22.4% (32/143) in cohort 2—the 400 days of this period yielded 40 enrolled participants, the greatest yield among all cohorts.

**Table 2:** Recruitment by cohort (N=2721).

Characteristic	Cohort 1 (initial enrollment; July 10, 2018, to June 19, 2019)		Cohort 2 (eligibility change; June 20, 2019, to January 20, 2020)			Cohort 3 (compensation change; January 21, 2020, to February 24, 2021)	
	n (%)	N	n (%)	N	n (%)		N
Screened	379 (100)	379	329 (100)	329	2013 (100)		2013
Eligible contacts (% of screened)	49 (12.9)	379	143 (43.5)	329	317 (15.7)		2013
Submitted proof of ART^a^ and VL^b^ (% of eligible)	16 (32.7)	49	44 (30.8)	143	58 (18.3)		317
Completed survey (% of eligible)	14 (28.6)	49	33 (23.1)	143	51 (16.1)		317
Enrolled (% of eligible)	11 (22.4)	49	32 (22.4)	143	40 (12.6)		317
Number of days	345 (100)	345	214 (100)	214	400 (100)		400

^a^ART: antiretroviral therapy.

^b^VL: viral load.

**Table 3:** Enrollment by cohort (n=83).

				Cohort 1 Enrolled (n=11), n (%)		Cohort 2 Enrolled (n=32), n (%)		Cohort 3 Enrolled (n=40), n (%)		Chi-square (*df*)			*P* value	
**Age (years)**											4.6 (4)			.33
	13-17		1 (9)		0 (0)		1 (3)							
	18-20		2 (18)		3 (9)		8 (20)							
	21-24		8 (73)		29 (91)		31 (78)							
**Race and ethnicity**											12.6 (6)			.05
	Black or African American		7 (64)		11 (34)		28 (70)							
	Hispanic		3 (27)		8 (25)		7 (18)							
	White		1 (9)		7 (22)		2 (5)							
	Other		0 (0)		6 (19)		3 (8)							
**Region**											7.4 (6)			.29
	Northeast		3 (27)		2 (6)		6 (15)							
	Midwest		3 (27)		5 (16)		4 (10)							
	South		4 (36)		20 (63)		20 (50)							
	West		1 (9)		5 (16)		10 (25)							
Metropolitan residence				8 (73)		24 (75)		29 (73)		0.1 (2)			.97	
**Sexual identity**											1.5 (4)			.82
	Gay		8 (73)		23 (72)		32 (80)							
	Straight		1 (9)		5 (16)		3 (8)							
	Bisexual		2 (18)		4 (13)		5 (13)							
**Gender identity**											4.0 (4)			.41
	Man		9 (82)		23 (72)		33 (83)							
	Woman		2 (18)		4 (13)		5 (13)							
	Nonbinary		0 (0)		5 (16)		2 (5)							
**Recruitment source**											1.3 (2)			.52
	**Paid advertisement**		8 (73)		17 (53)		23 (58)							
		Social media (Facebook)	3 (27)		9 (28)		8 (20)							
		Networking apps (Grindr and Jack’d)	5 (46)		8 (25)		15 (38)							
	**Unpaid recruitment**		3 (27)		15 (47)		17 (43)							
		Text in	1 (9)		3 (9)		0 (0)							
		Northwestern University	0 (0)		1 (3)		0 (0)							
		Online Master Screener or website	1 (9)		3 (9)		8 (20)							
		Other source	1 (9)		8 (25)		9 (23)							

**Table 4:** Recruitment by recruitment source (N=2721).

Characteristic	Paid recruitment, n (%)							Unpaid recruitment, n (%)		
	Social media (Facebook)			Networking apps (Grindr and Jack’d)			n (%)			N
	n (%)	N	n (%)		N					
Screened	476 (100)	476	1844 (100)		1844	401 (100)			401	
Eligible contacts (% of screened)	113 (23.7)	476	246 (13.34)		1844	150 (37.4)			401	
Received ART^a^ and VL^b^ (% of eligible contacts)	27 (23.9)	113	49 (19.9)		246	52 (34.7)			150	
Completed survey (% of received ART and VL)	20 (74.1)	27	33 (67.3)		49	36 (69.2)			52	
Enrolled (% of eligible contacts)	20 (17.7)	113	28 (11.4)		246	35 (23.3)			150	

^a^ART: antiretroviral therapy.

^b^VL: viral load.

**Table 5:** Enrollment by recruitment source (n=83).

		Enrolled by Social Media (Facebook; n=20), n (%)	Enrolled by Networking Apps (Grindr and Jack’d; n=28), n (%)		Enrolled by Unpaid Recruitment (n=35), n (%)		Chi-square (*df*)			*P* value	
**Age (years)**								5.6 (4)			.23
	13-17	0 (0)	0 (0)		2 (6)						
	18-20	2 (10)	3 (11)		8 (23)						
	21-24	18 (90)	25 (89)		25 (71)						
**Race and ethnicity**								3.3 (6)			.78
	Black or African American	11 (55)	16 (57)		19 (54)						
	Hispanic	5 (25)	5 (18)		8 (23)						
	White	3 (15)	2 (7)		5 (14)						
	Other	1 (5)	5 (18)		3 (89)						
**Region**								6.3 (6)			.39
	Northeast	2 (10)	6 (21)		3 (9)						
	Midwest	1 (5)	5 (18)		6 (17)						
	South	13 (65)	14 (50)		17 (49)						
	West	4 (20)	3 (11)		9 (26)						
Metropolitan residence		12 (60)	25 (89)		24 (69)		5.9 (2)			.05	
**Sexual identity**								5.4 (4)			.25
	Gay	15 (75)	25 (89)		23 (66)						
	Straight	3 (15)	1 (4)		5 (14)						
	Bisexual	2 (10)	2 (7)		7 (20)						
**Gender identity**								3.8 (4)			.43
	Man	15 (75)	24 (86)		26 (74)						
	Woman	4 (20)	1 (4)		6 (17)						
	Nonbinary	1 (5)	3 (11)		3 (9)						
**Cohort**								2.6 (4)			.62
	Cohort 1: initial enrollment	3 (15)	5 (18)		3 (9)						
	Cohort 2: SRA^a^ eligibility	9 (45)	8 (29)		15 (43)						
	Cohort 3: compensation increase	8 (40)	15 (54)	17 (49)							

^a^SRA: self-reported antiretroviral therapy adherence.

These data fail to account for rescreening efforts, whereby previously obtained contacts were screened again to assess eligibility after protocol changes. Duplicate screener responses were omitted by comparison of demographic data and IP addresses, with the latest eligible response kept where applicable. Passive recruitment in the form of links posted to various sites and distributed via hand cards helped to obtain eligible participants; dedicated advertisements and pooled recruitment surveys linked to paid advertisements on social media or networking apps also contributed substantially to the screened sample. Owing to the diversity of passive recruitment sources and paid recruitment efforts, the calculation of advertisement cost per enrollee is not possible.

### Enrollment Models

Table 6 and Multimedia Appendix 1 show the odds of enrollment of participants in the study (n=83) among eligible contacts (n=581). Those with most recent VL tests >6 months ago were half as likely to enroll (odds ratio [OR] 0.45, 95% CI 0.21-0.94). As expected, those with SRA between 50% and 80% were statistically significant and more likely to enroll than those with SRA >80% (50%: OR 2.72, 95% CI 1.41-5.26; *P*<.001; 60%: OR 2.44, 95% CI 1.24-4.81; *P*=.01; 70%: OR 2.83, 95% CI 1.43-5.60; *P*<.001; and 80%: OR 2.10, 95% CI 1.09-4.04; *P*=.03).

The odds of enrollment were not statistically significant with the cohort. Demographic identities—age category, binary racial or ethnic identity, region, metropolitan residence, gender identity, and sexual identity—were not associated with higher odds of enrollment. In addition, recruitment source was not found to be statistically significant with enrollment.

**Table 6:** Odds of full enrollment of participants in the study (n=83) by sociodemographic and behavioral characteristics among eligible participants (n=581).

Characteristics		OR^a^ (95% CI)	*P* value
**Age (years; ref^b^: 21-24)**			
	15-17	1.06 (0.35-3.27)	.92
	18-20	0.88 (0.56-1.39)	.59
**Race and ethnicity (ref: White)**			
	Black	0.76 (0.44-1.32)	.33
	Hispanic	0.76 (0.42-1.37)	.36
	Other	0.74 (0.36-1.52)	.41
**Region (ref: Northeast)**			
	Midwest	1.02 (0.53-1.96)	.96
	South	1.15 (0.67-1.97)	.61
	West	1.17 (0.62-2.20)	.62
Metropolitan residence		1.10 (0.72-1.70)	.66
**Gender identity (ref: man)**			
	Woman	2.08 (0.91-4.76)	.08
	Nonbinary	0.40 (0.16-1.00)	.05
**Sexual identity (ref: gay)**			
	Straight	1.19 (0.48-2.95)	.71
	Bisexual	0.97 (0.55-1.69)	.90
**Recruitment (ref: Reddit or other passive recruitment)**			
	Social media (paid)	0.77 (0.46-1.28)	.32
	Networking apps (paid)	0.71 (0.44-1.15)	.16
	Text-in, Sequential Multiple Assignment Randomized Trial Northwestern University referral (unpaid)	0.78 (0.34-1.78)	.56
	OMS^c^ or web page (unpaid)	1.36 (0.73-2.55)	.33
**Last VL^d^ test (ref: in the last month)**			
	2-3 months ago	0.94 (0.59-1.49)	.78
	4-6 months ago	0.84 (0.49-1.43)	.52
	>6 months ago	0.45^e^ (0.21-0.94)	.03
**Last VL result (ref: undetectable)**			
	At least 200 copies/mL	0.94 (0.51-1.73)	.83
	Unsure	0.93 (0.57-1.52)	.78
**Last detectable VL result (ref: in the last 3 months)**			
	4-6 months ago	1.35 (0.73-2.49)	.34
	7-12 months ago	1.01 (0.43-2.33)	.99
	>1 year ago	1.06 (0.53-2.12)	.87
	Never	1.16 (0.43-3.10)	.77
	Unsure	0.91 (0.44-1.88)	.79
**Date prescribed ART^f^ (ref: <6 months ago)**			
	6-12 months ago	1.11 (0.57-2.16)	.75
	>1 year ago	0.92 (0.54-1.58)	.77
**Self-reported adherence (ref: >80%)**			
	SRA^g^ 80%	2.10^e^ (1.09-4.04)	.03
	SRA 70%	2.83^h^ (1.43-5.60)	<.001
	SRA 60%	2.44^e^ (1.24-4.81)	.01
	SRA 50%	2.72^h^ (1.41-5.26)	<.001
	SRA 40%	1.85 (0.76-4.48)	.17
	SRA 30%	1.18 (0.50-2.78)	.70
	SRA 20%	1.53 (0.61-3.85)	.36
	SRA 10%	1.89 (0.77-4.59)	.16
	SRA 0%	1.58 (0.70-3.54)	.27
**Cohort (ref: cohort 1: initial enrollment)**			
	Cohort 2: SRA eligibility	0.96 (0.53-1.73)	.90
	Cohort 3: compensation increase	0.68 (0.39-1.20)	.18

^a^OR: odds ratio.

^b^ref: reference.

^c^OMS: Online Master Screener.

^d^VL: viral load.

^e^*P*<.05.

^f^ART: antiretroviral therapy.

^g^SRA: self-reported antiretroviral therapy adherence.

^h^*P*<.01.

## Discussion

### Principal Findings

This study explored recruitment and compensation strategies as well as the expansion of study eligibility criteria to include SRA-affected screening and enrollment milestones for youth living with HIV. The key findings include the eligibility rate and the number of enrolled participants increasing after expanding eligibility criteria to include SRA, being less successful than anticipated in using social media and dating app platforms for recruitment (likely because of the onset of the COVID-19 pandemic). Additionally, the study recruited the highest proportion of study enrollees after the OMS in cohort 3 from the website we launched to give providers information about the study and allow them to request recruitment materials that was sent to Ryan White Recipients on the HRSA listserve. These findings add to our knowledge on the use of paid advertisements on various social media platforms such as Facebook, Grindr, Jack’d, and Reddit as recruitment tools for youth living with HIV before and during the COVID-19 pandemic. They extend the literature by providing insight into the impact of expanding the eligibility criteria to include SRA during enrollment.

Facebook was the only source of paid advertisements to recruit participants in cohorts 1 and 2. For cohort 2, the eligibility criteria were expanded to include SRA of ≤80% in the past 30 days instead of exclusively providing proof of VL ≥200 copies/mL in blood specimens. The eligibility rate more than doubled, and the number of enrolled participants tripled in this cohort. Self-reported adherence using VASs has been found to be strongly associated with viral suppression in adolescent populations [32]. Although self-reported medication adherence may overestimate actual adherence and can be vulnerable to social desirability or recall bias [33,34], studies have found that participants reporting ≤80% SRA are statistically significant and less likely to achieve viral suppression [35]. Another meta-analysis found a robust pattern of association between SRA and VL and that SRA was statistically significant and was related to adherence, as assessed by other indirect measures [36]. As SRA is far easier for the participant to report than having to physically collect baseline VL data and submit it at enrollment, it may be a better measure to use when recruiting a sample of virally unsuppressed youth living with HIV. The efficiency of paid Facebook advertisements to collect screener responses that resulted in enrollment increased from 15% in cohort 1 to 45% in cohort 2 (Table 5), mirroring the general trends in eligibility increase for this cohort. As eligibility criteria were the only change in paid advertisement recruitment for this cohort, it is possible that the relative ease of submitting SRA information in comparison with having to acquire and submit VL data may be responsible for the increased eligibility of participants enrolled from paid screener advertisements.

The only additional source of recruitment for cohort 2 was referrals from the SMART Northwestern University study. Although Facebook yielded 9 participants in cohort 2 (45% of all enrolled in cohort 2), SMART Northwestern University referrals yielded only 1 enrollee (3% of those enrolled in cohort 2). This is potentially because the SMART Northwestern University study was a sexual health education study for adolescent MSM broadly, not youth living with HIV specifically. Although there is an overlap between the youth living with HIV and MSM populations, it may be helpful for future studies seeking to enroll youth living with HIV to obtain referrals from other studies that focus on adolescents who are HIV positive to recruit the target population.

There is little doubt that the initial spread of COVID-19 during cohort 3 affected recruitment, although the geographical distribution of this national sample and the varied regional transmission rates of COVID-19 render the effect unclear and unquantifiable. Cohort 3 retained an SRA of ≤80% as an eligibility criterion, but paid advertisements were also posted on dating apps and social media platforms such as Grindr, Jack’d, and Reddit in addition to Facebook to recruit participants, and compensation was increased to reflect recognition of participant burden. The overall number of screeners increased after posting these advertisements, although the proportion of eligible contacts, especially those who provided suitable contact information from paid advertisements, was lower. We did not find any statistically significant correlation between the increased compensation amounts and the number of participants that enrolled. However, as previously mentioned, the COVID-19 pandemic also began 2 months into the enrollment of cohort 3, so it is unclear as to how that also affected enrollment.

Grindr has generally been found to be particularly effective in reaching a large number of potential MSM participants in a short period [30]. However, enrollment for this cohort was paused for 2 months because of the start of the COVID-19 pandemic. Despite the restoration of recruitment through dating apps, people were using dating apps less frequently, in a significantly distinct manner than before, or not using them at all. McKay et al [37] found that between April 10 and May 10, 2020, 53% of the gay and bisexual men who participated in the study reported using dating apps or hookup sites or both less than they did before the start of the pandemic. Sanchez et al [38] found that many participants from their sample of MSM surveyed in April 2020 also had decreased use of dating or hookup apps, but many participants had unchanged use and a substantial proportion reported increased use to connect with other men but not to meet them. App users reported using apps to talk, pass the time, and get to know people but not immediately hook up, especially during early quarantine. Individuals may engage in preventative behaviors such as reducing their number of casual sex partners not only as a way to reduce their individual risk but also the risk of their social group and community [39]. As these apps are primarily designed to facilitate sexual interactions, it is possible that youth living with HIV who are more concerned about COVID-19 risk used the apps less to avoid engaging in behaviors that could increase transmission risk for themselves and their community. Even if potential contacts were using dating apps regularly, it is possible that not using the apps to meet up with people changed how they were engaging with the content on the apps such as study advertisements, which may explain the discrepancy in recruitment success compared with that of other studies.

For cohort 3, the highest proportion of enrolled screeners after the OMS came from the website created for providers and patients in Ryan White programs to download recruitment materials. Our approach was unique because we downloaded the Ryan White programs listserve from the HRSA website, created and implemented our website in cohort 2, sent out the link and a form to request recruitment materials to the contacts on the listserve, and later providers started to download and distribute the materials directly. The referrals provided the highest number of enrollees when compared proportionally across sources, which may be because of providers’ familiarity with the study eligibility requirements. It is unlikely that providers would refer to screeners who would be ineligible owing to criteria such as age, HIV status, VL, or SRA, as opposed to paid social media advertisements that cannot target potential participants with such specificity. It is also possible that participants referred by providers were more likely to enroll because they were already engaged in health care and that participants reached by web-based advertisements may not have been closely affiliated with care providers. Klassen et al [40] suggested that regular contact with health care facilities and face-to-face interactions with trusted sources such as health care providers may be critical in successfully recruiting participants who are HIV positive. Another study found success in partnering with Ryan White Case Managers to collect data from people with HIV, especially those who are hard to reach, disenfranchised, or underrepresented [41]. Leveraging the relationship between health care providers and youth living with HIV may yield a more specific, targeted, and efficient avenue to recruit a diverse pool of eligible participants, especially using the HRSA Ryan White HIV/AIDS Program’s comprehensive system of care and treatment [29].

In all cohorts, respondents from Facebook were somewhat less likely to be found eligible and submit contact information, although this was not statistically significant. Although Facebook has historically been a site that has significantly improved the engagement, screening, and enrollment of young sexual and gender minority adolescents [42,43], which overlaps with the population of youth living with HIV, we did not find paid screeners on Facebook to statistically significant increase participant enrollment. Although social media has also been successfully used to recruit participants either through advertisements [11] or direct recruitment [44], it is possible that the COVID-19 pandemic altered the way in which youth living with HIV used social media platforms, rendering them less effective for recruiting and enrolling eligible participants, despite the increased number of screeners.

After the eligibility criteria were expanded to include SRA, the eligibility and enrollment rates increased. As the pandemic evolves, using an SRA of ≤80% as an eligibility criterion instead of requiring proof of VL ≥200 copies/mL at baseline can be a useful tool, as it would be unethical to ask participants to leave their homes to obtain VL results for the study and risk potential exposure if the COVID-19 case rates and local transmission levels are high. Broadening eligibility criteria may be useful in recruiting those adolescents who are not adherent to assessment procedures or treatment dosing schedules before the randomization and initiation of the actual treatment [6] and, thus, making clinical trials more representative of maximizing the generalizability of results.

### Strengths and Limitations

There were several limitations to our data collection and analysis. First, the COVID-19 pandemic’s concurrence with cohort 3’s study enrollment complicates drawing conclusions about the effectiveness of dating apps or increased compensation as strategies to recruit youth living with HIV. COVID-19 likely changed how youth living with HIV relate to web-based spaces in ways that we were unable to measure or account for. Future research is needed to examine the effectiveness of dating apps as a recruitment tool for youth living with HIV during the COVID-19 pandemic as well as the effectiveness of compensation as an intervention for recruitment and enrollment. An additional limitation of using dating apps such as Grindr and Jack’d is that participants must be aged ≥18 years to use them. Testing the addition of bonuses for certain activities or examining the compensation elements most strongly associated with enrollment and retention can provide more clarity about the most effective degrees and types of compensation. In addition, we were unable to calculate the cost per enrollee because the paid advertisements contained links to surveys that screened for multiple studies, not just SMART, and included graphics that may or may not have been directly targeting youth living with HIV, which would affect the response rate. Finally, each platform that hosted advertisements had a different mechanism for advertisement display and pay rate for the type of advertisements; therefore, we do not have data on which types of advertisements received the most clicks, were most efficient at enrolling participants, or were most cost-efficient. A more comprehensive examination of the advertisement types and platforms that host them can provide more insight into which ones are the most cost-efficient for enrollment.

### Conclusions

Overall, despite the limitations of the study and the absence of statistically significant findings, this study provided insight into how recruitment strategies such as social media advertisements and provider referrals as well as expanding eligibility criteria affected screening and enrollment numbers. As the COVID-19 pandemic evolves and the use of and engagement with social media and dating apps among youth living with HIV changes, these platforms should continue to be investigated as potential recruitment tools. Using a wide variety of recruitment strategies including social media and provider referrals through the Ryan White program, increasing compensation amounts, and including SRA in enrollment criteria should continue to be studied with respect to their ability to successfully recruit and enroll eligible participants.

## Appendix

Multimedia Appendix 1 Sociodemographic and behavioral differences in completing milestone from eligibility to full enrollment (ie, submitting antiretroviral therapy and viral load and completing baseline survey).

## Abbreviations

ART: antiretroviral therapy
ATN: Adolescent Medicine Trials Network for HIV/AIDS Interventions
HRSA: Health Resources and Services Administration
IRB: institutional review board
MSM: men who have sex with men
OMS: Online Master Screener
OR: odds ratio
SMART: Sequential Multiple Assignment Randomized Trial
SRA: self-reported antiretroviral therapy adherence
SRV: Subject Recruitment Venue
VAS: visual analog scale
VL: viral load

## References

[1] National Center for HIV, Viral Hepatitis, STD, and TB Prevention. Centers for Disease Control and Prevention.

[2] Agwu AL, Yusuf HE, D'Angelo L, Rathore M, Marchesi J, Rowell J, Smith R, Toppins J, Trexler C, Carr R, Johnson B, Selden AK, Mahmoudi S, Black S, Guadamuz J, Huettner S, Trent M (2020). Recruitment of youth living with HIV to optimize adherence and virologic suppression: testing the design of technology-based community health nursing to improve antiretroviral therapy (ART) clinical trials. JMIR Res Protoc.

[3] Fernandez MI, Harper GW, Hightow-Weidman LB, Kapogiannis BG, Mayer KH, Parsons JT, Rotheram-Borus MJ, Seña AC, Sullivan PS (2021). Research priorities to end the adolescent HIV epidemic in the United States: viewpoint. JMIR Res Protoc.

[4] Hunt JR, White E (1998). Retaining and tracking cohort study members. Epidemiol Rev.

[5] Navarra AD, Handschuh C, Hroncich T, Jacobs SK, Goldsamt L (2020). Recruitment of US adolescents and young adults (AYA) into human immunodeficiency virus (HIV)-related behavioral research studies: a scoping review. Curr HIV/AIDS Rep.

[6] DiClemente R, Ruiz M, Sales J (2010). Barriers to adolescents' participation in HIV biomedical prevention research. J Acquir Immune Defic Syndr.

[7] Sagrestano LM, Clay J, Finerman R, Gooch J, Rapino M (2014). Transportation vulnerability as a barrier to service utilization for HIV-positive individuals. AIDS Care.

[8] Parker JN, Hunter AS, Bauermeister JA, Bonar EE, Carrico A, Stephenson R (2021). Comparing social media and in-person recruitment: lessons learned from recruiting substance-using, sexual and gender minority adolescents and young adults for a randomized control trial. JMIR Public Health Surveill.

[9] Grov C (2012). HIV risk and substance use in men who have sex with men surveyed in bathhouses, bars/clubs, and on Craigslist.org: venue of recruitment matters. AIDS Behav.

[10] Parsons JT, Vial AC, Starks TJ, Golub SA (2013). Recruiting drug using men who have sex with men in behavioral intervention trials: a comparison of internet and field-based strategies. AIDS Behav.

[11] Iribarren SJ, Ghazzawi A, Sheinfil AZ, Frasca T, Brown W, Lopez-Rios J, Rael CT, Balán IC, Crespo R, Dolezal C, Giguere R, Carballo-Diéguez A (2018). Mixed-method evaluation of social media-based tools and traditional strategies to recruit high-risk and hard-to-reach populations into an HIV prevention intervention study. AIDS Behav.

[12] Zlotorzynska M, Bauermeister JA, Golinkoff JM, Lin W, Sanchez TH, Hightow-Weidman L (2021). Online recruitment of youth for mHealth studies. Mhealth.

[13] Thornton L, Batterham PJ, Fassnacht DB, Kay-Lambkin F, Calear AL, Hunt S (2016). Recruiting for health, medical or psychosocial research using Facebook: systematic review. Internet Interv.

[14] Vermund SH, Hamilton EL, Griffith SB, Jennings L, Dyer TV, Mayer K, Wheeler D (2018). Recruitment of underrepresented minority researchers into HIV prevention research: the HIV prevention trials network scholars program. AIDS Res Human Retroviruses.

[15] HIV and Women. HIVinfo. NIH.gov.

[16] Blümle A, Schandelmaier S, Oeller P, Kasenda B, Briel M, von Elm E, DISCO study group (2016). Premature discontinuation of prospective clinical studies approved by a research ethics committee - a comparison of randomised and non-randomised studies. PLoS One.

[17] Williams RJ, Tse T, DiPiazza K, Zarin DA (2015). Terminated trials in the clinicaltrials.gov results database: evaluation of availability of primary outcome data and reasons for termination. PLoS One.

[18] Nahum-Shani I, Qian M, Almirall D, Pelham WE, Gnagy B, Fabiano GA, Waxmonsky JG, Yu J, Murphy SA (2012). Experimental design and primary data analysis methods for comparing adaptive interventions. Psychol Methods.

[19] Belzer ME, MacDonell KK, Ghosh S, Naar S, McAvoy-Banerjea J, Gurung S, Cain D, Fan CA, Parsons JT (2018). Adaptive antiretroviral therapy adherence interventions for youth living with HIV through text message and cell phone support with and without incentives: protocol for a sequential multiple assignment randomized trial (SMART). JMIR Res Protoc.

[20] Finitsis DJ, Pellowski JA, Huedo-Medina TB, Fox MC, Kalichman SC (2016). Visual analogue scale (VAS) measurement of antiretroviral adherence in people living with HIV (PLWH): a meta-analysis. J Behav Med.

[21] Horvath KJ, Amico KR, Erickson D, Ecklund AM, Martinka A, DeWitt J, McLaughlin J, Parsons JT (2018). Thrive with me: protocol for a randomized controlled trial to test a peer support intervention to improve antiretroviral therapy adherence among men who have sex with men. JMIR Res Protoc.

[22] Huang GD, Bull J, Johnston McKee K, Mahon E, Harper B, Roberts JN, CTTI Recruitment Project Team (2018). Clinical trials recruitment planning: a proposed framework from the clinical trials transformation initiative. Contemp Clin Trials.

[23] Kempf L, Goldsmith JC, Temple R (2018). Challenges of developing and conducting clinical trials in rare disorders. Am J Med Genet A.

[24] Nagy SM, Butame SA, Todd L, Sheffler JL, Budhwani H, Fernandez MI, MacDonell K, Naar S (2022). Barriers and facilitators to implementing a motivational interviewing-based intervention: a multi-site study of organizations caring for youth living with HIV. AIDS Care.

[25] Kapogiannis B, Koenig L, Xu J, Mayer K, Loeb J, Greenberg L, Monte D, Banks-Shields M, Fortenberry JD, Adolescent Medicine Trials Network for HIV/AIDS Interventions (2020). The HIV continuum of care for adolescents and young adults attending 13 urban US HIV care centers of the NICHD-ATN-CDC-HRSA SMILE collaborative. J Acquir Immune Defic Syndr.

[26] Lee S, Kapogiannis BG, Allison S (2019). Improving the youth HIV prevention and care continuums: the adolescent medicine trials network for HIV/AIDS interventions. JMIR Res Protoc.

[27] Budhwani H, Robles G, Starks TJ, MacDonell KK, Dinaj V, Naar S (2021). Healthy choices intervention is associated with reductions in stigma among youth living with HIV in the United States (ATN 129). AIDS Behav.

[28] Naar S, Parsons JT, Stanton BF (2019). Adolescent trials network for HIV-AIDS scale it up program: protocol for a rational and overview. JMIR Res Protoc.

[29] Ryan White HIV/AIDS recipients and sub–recipients. Health Resources & Services Administration.

[30] Mass texting service. Trumpia Homepage.

[31] Starks TJ, Skeen SJ, Scott Jones S, Gurung S, Millar BM, Ferraris C, Ventuneac A, Parsons JT, Sparks MA (2022). Effectiveness of a combined motivational interviewing and cognitive behavioral intervention to reduce substance use and improve HIV-related immune functioning. AIDS Behav.

[32] Dziva Chikwari C, Ferrand R, Simms V (2017). Association between self-reported adherence and HIV viral load suppression among older children and adolescents. J Acquir Immune Defic Syndr.

[33] Gold DT (2006). Medication adherence. J Manag Care Spec Pharm.

[34] Williams AB, Amico KR, Bova C, Womack JA (2013). A proposal for quality standards for measuring medication adherence in research. AIDS Behav.

[35] O'Halloran Leach E, Lu H, Caballero J, Thomas JE, Spencer EC, Cook RL (2021). Defining the optimal cut-point of self-reported ART adherence to achieve viral suppression in the era of contemporary HIV therapy: a cross-sectional study. AIDS Res Ther.

[36] Simoni JM, Kurth AE, Pearson CR, Pantalone DW, Merrill JO, Frick PA (2006). Self-report measures of antiretroviral therapy adherence: a review with recommendations for HIV research and clinical management. AIDS Behav.

[37] McKay T, Henne J, Gonzales G, Gavulic KA, Quarles R, Gallegos SG (2021). Sexual behavior change among gay and bisexual men during the first COVID-19 pandemic wave in the United States. Sex Res Social Policy.

[38] Sanchez TH, Zlotorzynska M, Rai M, Baral SD (2020). Characterizing the impact of COVID-19 on men who have sex with men across the United States in April, 2020. AIDS Behav.

[39] Gupta SK, Dellucci TV, Stewart JL, Starks TJ (2021). Perceived risk, optimistic bias, and united action: a socio-ecological examination of COVID-19 prevention behaviors among sexual minority men. Psychol Sex Orientat Gend Divers.

[40] Klassen KM, Millard T, Stout J, McDonald K, Dodson S, Osborne RH, Battersby MW, Fairley CK, Kidd MR, McMahon J, Baker D, Elliott JH (2019). Recruiting people with HIV to an online self-management support randomised controlled trial: barriers and facilitators. Sex Health.

[41] Lockhart E, Turner D, Galea JT, Marhefka SL (2022). Considerations for partnering with Ryan White Case Managers to create equitable opportunities for people with HIV to participate in research. PLoS One.

[42] Baker R, Zlotorzynska M, Knopf AS (2020). 76. Use of social media for the recruitment and engagement of adolescent sexual and gender minorities in HIV research studies. J Adolesc Health.

[43] Shao W, Guan W, Clark MA, Liu T, Santelices C, Cortés DE, Merchant RC (2015). Variations in recruitment yield, costs, speed and participant diversity across internet platforms in a global study examining the efficacy of an HIV/AIDS and HIV testing animated and live-action video among English- or Spanish-speaking internet or social media users. Digit Cult Educ.

[44] Ocasio MA, Fernandez MI, Joseph JM, Rezai R, CARES Team Atn (2021). Engaging sexual and gender minority youth in HIV interventions through gay dating apps: recruitment protocol. JMIR Res Protoc.

