# Supplementary material for: Examining Recruitment Strategies in the Enrollment Cascade of Youth Living With HIV: Descriptive Findings From a Nationwide Web-Based Adherence Protocol
Source: JMIR Form Res. 2023 Apr 12;7:e40077. doi: 10.2196/40077 (PMC10131637; doi:10.2196/40077)
Supplement: Multimedia Appendix 1 [file formative_v7i1e40077_app1.docx]

**Multimedia Appendix 1.** Sociodemographic and behavioral differences in completing milestone from eligibility to full enrollment (ie, submitting antiretroviral therapy and viral load and completing baseline survey).

| Characteristic | Eligible (n=581) of all screened (n=2721) |  | Eligible contacts (n=509) of all eligible (n=581) |  | ART and VL received (n=128) of eligible contacts (n=509) |  | Completed survey (n=97) of eligible contacts (n=509) |  |
| --- | --- | --- | --- | --- | --- | --- | --- | --- |
|  | n (%) | Chi-square (df), *P* value | n (%) | Chi-square (df), *P* value | n (%) | Chi-square (df), *P* value | n (%) | Chi-square (df), *P* value |
| Age |  | 390.75 (2), P<.001 |  | 1.48 (2), P=.48 |  | 1.71 (2), P=.43 |  | 0.72 (2), P=.70 |
| 13-17 | 13 (2.2%) |  | 10 (2.0%) |  | 2 (1.6%) |  | 2 (2.1%) |  |
| 18-20 | 108 (18.6%) |  | 94 (18.5%) |  | 19 (14.8%) |  | 15 (15.5%) |  |
| 21-24 | 460 (79.2%) |  | 405 (79.6%) |  | 107 (83.6%) |  | 80 (82.7%) |  |
| Race/Ethnicity |  | 75.89 (3), P<.001 |  | 2.04 (3), P=.56 |  | 1.28 (3), P=.73 |  | 1.24 (3), P=.74 |
| Black/African American | 328 (56.5%) |  | 286 (56.2%) |  | 69 (53.9%) |  | 55 (56.7%) |  |
| Hispanic | 121 (20.8%) |  | 107 (21.0%) |  | 26 (20.3%) |  | 20 (20.6%) |  |
| White | 61 (10.5%) |  | 51 (10.0%) |  | 16 (12.5%) |  | 12 (12.4%) |  |
| Other | 71 (12.2%) |  | 65 (12.8%) |  | 17 (13.3%) |  | 10 (10.3%) |  |
| Region |  | 4.76 (3), P=.19 |  | 0.96 (3), P=.81 |  | 2.28 (3), P=.52 |  | 1.72 (3), P=.63 |
| Northeast | 101 (17.4%) |  | 87 (17.1%) |  | 20 (15.6%) |  | 13 (13.4%) |  |
| Midwest | 91 (15.7%) |  | 82 (16.1%) |  | 16 (12.5%) |  | 14 (14.4%) |  |
| South | 283 (48.7%) |  | 246 (48.3%) |  | 66 (51.6%) |  | 50 (51.5%) |  |
| West | 106 (18.2%) |  | 94 (18.5%) |  | 26 (20.3%) |  | 20 (20.6%) |  |
|  |  | 5.12 (1), P=.02 |  | 0.53 (1), P=.47 |  | 0.08 (1), P=.78 |  | 0.18 (1), P=.68 |
| Metropolitan residence | 432 (74.4%) |  | 381 (74.9%) |  | 97 (75.8%) |  | 71 (73.2%) |  |
| Sexual Identity |  | 10.29 (3), P=.02 |  | 3.02 (3), P=.39 |  | 7.00 (3), P=.07 |  | 9.18 (3), P=.03 |
| Gay | 437 (75.2%) |  | 388 (76.2%) |  | 100 (78.1%) |  | 76 (78.4%) |  |
| Straight | 36 (6.2%) |  | 30 (5.9%) |  | 12 (9.4%) |  | 10 (10.3%) |  |
| Bisexual | 84 (14.5%) |  | 72 (14.1%) |  | 14 (10.9%) |  | 11 (11.3%) |  |
| Queer, other sexual identity | 24 (4.1%) |  | 19 (3.7%) |  | 2 (1.6%) |  | 0 (0.0%) |  |
| Gender Identity |  | 13.15 (2), P=.001 |  | 5.28 (2), P=.07 |  | 3.16 (2), P=.21 |  | 7.86 (2), P=.02 |
| Man | 495 (85.2%) |  | 437 (85.9%) |  | 106 (82.8%) |  | 78 (80.4%) |  |
| Woman | 37 (6.4%) |  | 28 (5.5%) |  | 11 (8.6%) |  | 11 (11.3%) |  |
| Nonbinary | 49 (8.4%) |  | 44 (8.6%) |  | 11 (8.6%) |  | 8 (8.2%) |  |
| Recruitment Source |  | 104.28 (1), P<.001 |  | 4.07 (1), P=.04^a^ |  | 10.24 (1), P<.001^a^ |  | 5.43 (1), P=.02^a^ |
| Paid Recruitment | 418 (71.9%) |  | 359 (70.5%) |  | 76 (59.4%) |  | 59 (60.8%) |  |
| Social Media (Facebook) | 127 (21.9%) |  | 102 (20.0%) |  | 27 (21.1%) |  | 20 (20.6%) |  |
| Networking Apps (Grindr, Jack'd) | 276 (47.5%) |  | 246 (48.3%) |  | 49 (38.3%) |  | 39 (40.2%) |  |
| Reddit | 15 (2.6%) |  | 11 (2.2%) |  | 0 (0.0%) |  | 0 (0.0%) |  |
| Unpaid Recruitment | 163 (28.1%) |  | 150 (29.5%) |  | 52 (40.6%) |  | 38 (39.2%) |  |
| Text-in | 21 (3.6%) |  | 21 (4.1%) |  | 8 (6.3%) |  | 4 (4.1%) |  |
| Northwestern University | 6 (1.0%) |  | 5 (1.0%) |  | 2 (1.6%) |  | 1 (1.0%) |  |
| Online Master Screener, Website | 55 (9.5%) |  | 48 (9.4%) |  | 18 (14.1%) |  | 12 (12.4%) |  |
| Other source | 81 (13.9%) |  | 76 (14.9%) |  | 24 (18.8%) |  | 21 (21.6%) |  |
| Most Recent VL Test |  | 383.32 (4), P<.001 |  | 12.16 (4), P=.02 |  | 7.58 (4), P=.11 |  | 7.66 (4), P=.11 |
| Less than 1 month ago | 162 (27.9%) |  | 147 (28.9%) |  | 43 (33.6%) |  | 27 (27.8%) |  |
| 2-3 months ago | 213 (36.7%) |  | 178 (35.0%) |  | 46 (35.9%) |  | 40 (41.2%) |  |
| 4-6 months ago | 130 (22.4%) |  | 117 (23.0%) |  | 29 (22.7%) |  | 25 (25.8%) |  |
| 7-12 months ago | 54 (9.3%) |  | 51 (10.0%) |  | 10 (7.8%) |  | 4 (4.1%) |  |
| More than 1 year ago | 22 (3.8%) |  | 16 (3.1%) |  | 0 (0.0%) |  | 1 (1.0%) |  |
| Most Recent VL Result |  | 604.86 (2), P<.001 |  | 2.88 (2), P=.24 |  | 1.79 (2), P=.41 |  | 14.39 (2), P<.001 |
| Undetectable,<200 copies/mL | 169 (29.1%) |  | 152 (29.9%) |  | 44 (34.4%) |  | 44 (45.4%) |  |
| Detectable, at least 200 copies/mL | 159 (27.4%) |  | 142 (27.9%) |  | 32 (25.0%) |  | 24 (24.7%) |  |
| Unsure | 253 (43.5%) |  | 215 (42.2%) |  | 52 (40.6%) |  | 29 (29.9%) |  |
| Last Detectable VL Test |  | 392.90 (5), P<.001 |  | 6.03 (5), P=.30 |  | 2.22 (5), P=.82 |  | 8.18 (5), P=.15 |
| Less than 3 months ago | 172 (29.6%) |  | 151 (29.7%) |  | 39 (30.5%) |  | 27 (27.8%) |  |
| 4-6 months ago | 80 (13.8%) |  | 73 (14.3%) |  | 18 (14.1%) |  | 18 (18.6%) |  |
| 7-12 months ago | 48 (8.3%) |  | 42 (8.3%) |  | 11 (8.6%) |  | 10 (10.3%) |  |
| More than 1 year ago | 97 (16.7%) |  | 88 (17.3%) |  | 25 (19.5%) |  | 22 (22.7%) |  |
| Never detectable | 28 (4.8%) |  | 26 (5.1%) |  | 8 (6.3%) |  | 4 (4.1%) |  |
| Unsure | 156 (26.9%) |  | 129 (25.3%) |  | 27 (21.1%) |  | 16 (16.5%) |  |
| ART Prescription |  | 379.16 (1), P<.001 |  | 0.18 (1), P=.67 |  | 7.88 (1), P=.005 |  | 6.06 (1), P=.01 |
| Formerly prescribed | 49 (8.4%) |  | 42 (8.3%) |  | 3 (2.3%) |  | 2 (2.1%) |  |
| Currently prescribed | 532 (91.6%) |  | 467 (91.7%) |  | 125 (97.7%) |  | 95 (97.9%) |  |
| First prescribed ART |  | 498.90 (2), P<.001 |  | 2.76 (2), P=.25 |  | 0.93 (2), P=.63 |  | 0.77 (2), P=.68 |
| 3-6 months ago | 77 (13.3%) |  | 63 (12.4%) |  | 15 (11.7%) |  | 11 (11.3%) |  |
| 7-12 months ago | 90 (15.5%) |  | 80 (15.7%) |  | 17 (13.3%) |  | 18 (18.6%) |  |
| More than 12 months ago | 414 (71.3%) |  | 366 (71.9%) |  | 96 (75.0%) |  | 68 (70.1%) |  |
| Self-Reported Adherence (SRA) |  | 278.04, P<.001^c^ |  | 1.19, P=.17b |  | 2.68, P=.10^b^ |  | 19.10, P<.001^b^ |
| SRA > 80% | 170 (29.3%) |  | 144 (28.3%) |  | 29 (22.7%) |  | 10 (10.3%) |  |
| SRA ≤ 80% | 411 (70.7%) |  | 365 (71.7%) |  | 99 (77.3%) |  | 87 (89.7%) |  |
| SRA=80% | 86 (3.2%) |  | 77 (15.1%) |  | 21 (16.4%) |  | 20 (20.6%) |  |
| SRA=70% | 67 (2.5%) |  | 58 (11.4%) |  | 16 (12.5%) |  | 19 (19.6%) |  |
| SRA=60% | 34 (1.2%) |  | 31 (6.1%) |  | 11 (8.6%) |  | 10 (10.3%) |  |
| SRA=50% | 57 (2.1%) |  | 57 (9.8%) |  | 20 (15.6%) |  | 16 (16.5%) |  |
| SRA=40% | 35 (1.3%) |  | 35 (6.0%) |  | 9 (7.0%) |  | 7 (7.2%) |  |
| SRA=30% | 42 (1.5%) |  | 42 (7.2%) |  | 7 (5.5%) |  | 3 (3.1%) |  |
| SRA=20% | 25 (0.9%) |  | 25 (4.3%) |  | 5 (3.9%) |  | 3 (3.1%) |  |
| SRA=10% | 21 (0.8%) |  | 21 (3.6%) |  | 4 (3.1%) |  | 4 (4.1%) |  |
| SRA=0% | 44 (1.6%) |  | 44 (7.6%) |  | 6 (4.7%) |  | 5 (5.2%) |  |
|  |  | 145.81 (2), P<.001 |  | 5.77 (2), P=.06 |  | 7.48 (2), P=.02 |  | 5.71 (2), P=.06 |
| Cohort 1: Initial Enrollment | 59 (10.2%) |  | 49 (9.6%) |  | 17 (13.3%) |  | 14 (14.4%) |  |
| Cohort 2: SRA Eligibility | 154 (26.5%) |  | 143 (28.1%) |  | 44 (34.4%) |  | 32 (33.0%) |  |
| Cohort 3: Compensation Increase | 368 (63.3%) |  | 317 (62.3%) |  | 67 (52.3%) |  | 51 (52.6%) |  |

a values compare aggregated paid vs unpaid recruitment

b values compare SRA > 80% vs SRA ≤ 80%

c values compare SRA > 80% vs SRA ≤ 80% and only those prescribed ART, n=1823
